# Preliminary Results of the “Capasquelet” Technique for Managing Femoral Bone Defects—Combining a Masquelet Induced Membrane and Capanna Vascularized Fibula with an Allograft

**DOI:** 10.3390/jpm11080774

**Published:** 2021-08-09

**Authors:** Alexis Combal, François Thuau, Alban Fouasson-Chailloux, Pierre-Paul Arrigoni, Marc Baud’huin, Franck Duteille, Vincent Crenn

**Affiliations:** 1 Clinique Chirurgicale Orthopédique et Traumatologique, CHU de Nantes, University Hospital of Nantes, 44093 Nantes, France; alexis.combal@chu-nantes.fr; 2 Service de Chirurgie Plastique et Reconstructrice, CHU de Nantes, University Hospital of Nantes, 44093 Nantes, France; francois.thuau@chu-nantes.fr (F.T.); franck.duteille@chu-nantes.fr (F.D.); 3 Service de Médecine Physique et de Réadaptation Locomotrice, CHU de Nantes, University Hospital of Nantes, 44093 Nantes, France; alban.fouassonchailloux@chu-nantes.fr; 4 Service de Radiologie, CHU de Nantes, University Hospital of Nantes, 44093 Nantes, France; pierrepaul.arrigoni@chu-nantes.fr; 5 Banque Multi-Tissus, CHU de Nantes, University Hospital of Nantes, 44093 Nantes, France; marc.baudhuin@chu-nantes.fr; 6 INSERM U1238—Faculté de Médecine 1 rue Gaston Veil, 44035 Nantes, France

**Keywords:** critical bone defect, vascularized fibula, Capanna technique, Masquelet induced membrane, intercalary reconstruction, bone tumor, ballistic trauma

## Abstract

We describe the preliminary results of a novel two-stage reconstruction technique for extended femoral bone defects using an allograft in accordance with the Capanna technique with an embedded vascularized fibula graft in an induced membrane according to the Masquelet technique. We performed what we refer to as “Capasquelet” surgery in femoral diaphyseal bone loss of at least 10 cm. Four patients were operated on using this technique: two tumors and two traumatic bone defects in a septic context with a minimum follow up of one year. Consolidation on both sides, when achieved, occurred at 5.5 months (4–7), with full weight-bearing at 11 weeks (8–12). The functional scores were satisfactory with an EQ5D of 63.3 (45–75). The time to bone union and early weight-bearing with this combined technique are promising compared to the literature. The osteoinductive role of the induced membrane could play a positive role in the evolution of the graft. Longer follow up and a larger cohort are needed to better assess the implications. Nonetheless, this two-stage technique appears to have ample promise, especially in a septic context or in adjuvant radiotherapy in an oncological context.

## 1. Introduction

Critical diaphyseal bone defects remain a surgical challenge, and several treatment methods have been described [1,2,3,4,5,6] (autograft, vascularized fibula, bone transport, diaphyseal endoprosthesis, etc.). The gold-standard indications are still a matter of debate, and the results are variable. Malignant bone tumors, with their specificities, such as chemotherapy and radiotherapy [7] or septic ballistic trauma, add complexity to this challenging management [7,8,9,10].

We describe a novel two-stage technique for reconstruction of extended femoral bone defects, combining the allograft technique with inlay of a vascularized fibula (i.e., “Capanna technique”) [11] and the induced membrane technique (i.e., “Masquelet technique”) [1,12]. We performed this innovative procedure in critical bone defects of at least 10 cm that were due to the fact of tumor pathology secondary to carcinologic resection or a traumatic event. The combination proposed by what we refer to as the “Capasquelet” technique provides several advantages: the biological chamber with the induced membrane prevents resorption of the graft and has an osteoinductive role [13,14,15,16]; it also limits septic risks, and it avoids a long one-stage surgery [17]. The vascularized fibula can be more than 20 cm in size, and it improves both intercalary allograft survival and bone union [18]; some of the autologous grafts are also impacted at the interfaces [19]. By combining these methods, the “Capasquelet” technique can pool these advantages: the contribution of a living graft through the vascularized fibula, associated with the primary mechanical strength of the allograft in an environment that encourages osteogenesis, and an autologous bone graft at the extremities.

This preliminary study describes this novel hybrid surgical technique. We assessed the bone healing, the time to full weight-bearing, and the complications in addition to conducting a functional score analysis.

## 2. Method

The Masquelet technique proposes a two-stage procedure combining induced membrane and cancellous autograft [12]. While the Capanna technique offers a hybrid reconstruction with a vascularized fibula embedded in an allograft [11]. With the “Capasquelet” procedure, we propose to combine these two methods. This two-stage surgery was performed on four patients in a similar manner. The time between the two surgical stages was decided on a case-by-case basis (17 weeks (8–24)). An angio-CT scan was systematically performed before the second stage to check the blood supply and to confirm the feasibility of performing the vascular anastomosis of the fibular graft.

### 2.1. Surgical Technique

#### 2.1.1. The First Stage

**Figure 1:**
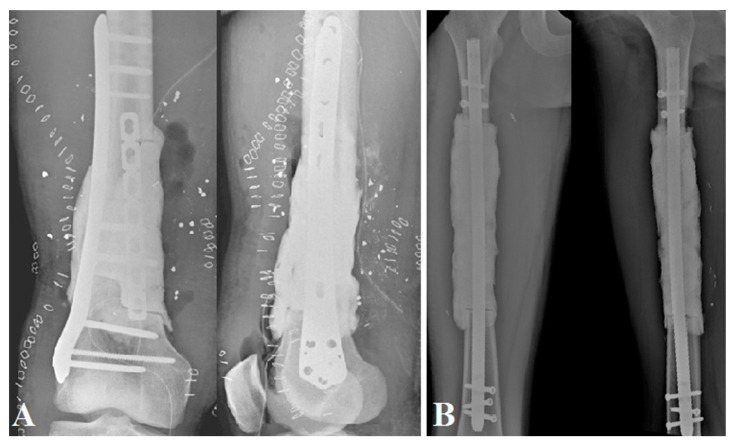
Postoperative X-ray AP and lateral view of the spacers: (**A**) a plate-enhanced spacer on a bone defect with step-cut in setting a septic ballistic trauma of the right distal femur (patient No. 4); (**B**) an intramedullary nail-enhanced spacer on a bone defect after an Ewing sarcoma resection.

The two patients with bone tumors underwent initial oncologic resection; the other patients had post-traumatic septic lesions that required debridement, irrigation, bacteriological sampling, and antibiotic therapy. The femur was prepared with a clear, clean cut in a healthy zone, if necessary, either transversally (*n* = 3) or in step-cut (*n* = 1). The aim of this first step was to model a cement spacer using high-viscosity Heraeus Palacos^®^ R + G cement (Hanau, Germany); it has to fill the bone loss as fully as possible. We preferred to oversize it somewhat in width if the soft tissue coverage was not an issue, as it increases reconstruction space and facilitates induced membrane closure for the second stage. The spacer was needed to cover the bone–host interfaces as recommended in the Masquelet technique [12]. It was enhanced on a locked intramedullary nailing, positioned back and forth (*n* = 3) or on a plate (*n* = 1) (Figure 1).

#### 2.1.2. Interstage Planification

The femoral allograft dimensions were determined to obtain a morphology that resembles that of the patient as much as possible; the diaphysis shaft width must allow the fibula to slide into it. We used cryopreserved allografts from the Nantes Multi-Tissue Bank, in compliance with the criteria of the French Agency of Biomedicine. The delay between the two stages depended on the indication and its requirements: the degree of tissue and skin healing or the antibiotic duration in trauma and adjuvant therapy planning in oncological situations.

#### 2.1.3. The Second Stage

The length and the rotational axis had to be carefully determined before spacer removal. The vascularized fibula graft required microsurgical expertise for harvesting and vascular anastomoses. The fibula graft could be ≥4 cm of the bone defect length. Graft harvesting could be undertaken from the contralateral limb by a second team with microsurgical skills in order to limit the operative time. The harvesting was performed with a tourniquet while ensuring that at least 8 cm [20] of the distal fibula was left in order to limit malleolar instability. A lateral surgical approach was used to raise the osseous flap. The fibula was mainly vascularized by an artery from the peroneal artery that entered the middle-third of the bone. The section must include this part of the bone. After identification of the artery, it was dissected to its origin from the tibio-fibular trunk [21,22].

**Figure 2:**
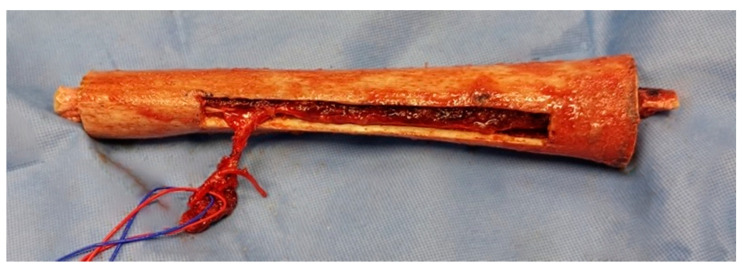
Vascularized fibula embedded in the femoral allograft before implantation. The allograft window makes vascular anastomosis possible. There was an extra 20 mm after each extremity of the fibula (patient No. 3).

The allograft was left for one hour in a warm physiological serum bath and then prepared with an oscillating saw in relation to the length of the bone defect; one team could be devoted to preparing the allograft (Figure 2). The microsurgical anastomosis was usually performed at the terminal branches of the profunda femoris artery or with an end-to-side anastomosis with the superficial femoral artery (Figure 2) [23].

The allograft was reamed to the diameter required (≥2 mm of the maximum diameter of the fibula). A bone window was created for the passage and preservation of the arteriovenous axis given the micro-anastomoses, oriented towards the vascular axis for anastomosis. The patient’s femur was also reamed, and the reaming material was preserved to perform a complementary autologous bone graft at the interfaces.

The composite graft was then placed in the correct orientation, while taking into account the rotational axes. The fibula had to be well-positioned to obtain an equivalent proximal and distal overlap of approximately 2 cm.

**Figure 3:**
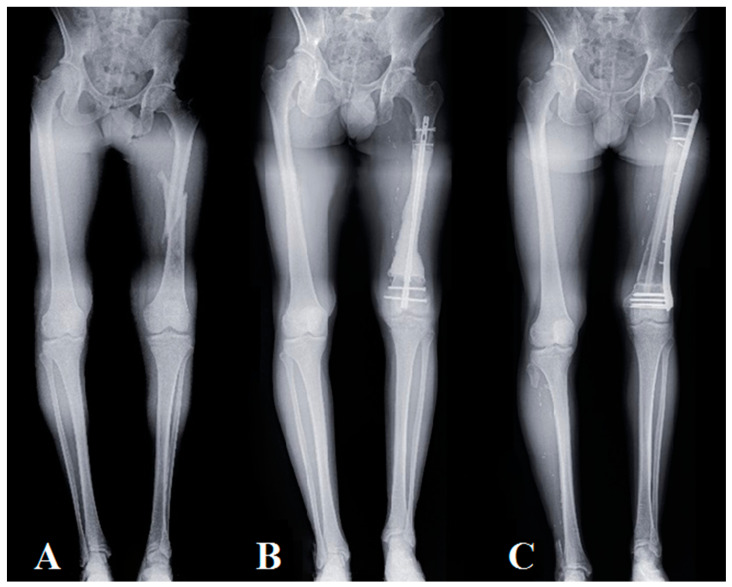
Progression of a Capasquelet technique reconstruction, using a pangonogram X-ray view, in a 28 year old patient with high-grade osteosarcoma (patient No. 2): (**A**) preoperative X-ray, fractured high-grade osteosarcoma with a length inequality of 80 mm; (**B**) postoperative X-ray of the 1st surgical stage after carcinologic resection and placement of the spacer on an intramedullary nail with a length inequality of 60 mm (post-T1 delay: 1 month); (**C**) postoperative X-ray at 1 year, residual lower limb inequality of 40 mm.

Stabilization was achieved with a large LCP plate; the graft was held in place by at least two single cortical screws. Finally, compression was applied at both interfaces after the autologous graft bone from the reaming was added (Figure 3).

#### 2.1.4. Postoperative Management

Early mobilization and progressive weight-bearing were allowed postoperatively. Strictly limited weight-bearing was prescribed for the first six weeks, with progressive full weight-bearing depending on the patient’s pain and condition.

### 2.2. Data Collection and Analysis

We performed the “Capasquelet” technique on four patients with bone defects of at least 10 cm. Two tumors and two traumatic lesions were operated on between 2018 and 2020 with a minimum follow up of one year. We collected the following data for each case: age, gender, BMI, characteristics of the defect, and surgical and postoperative data. Bone consolidation was assessed radiologically (postoperative X-rays were performed at least every three months until union). This criterion was defined by cortical union of at least 75%, as determined on standard X-rays. If the assessment of union was inconclusive on conventional X-rays, the union was assessed using computed tomography (CT) [24]. Surgical intervention to facilitate union of osseous junctions in Henderson type 2 complications, at least six months after the primary surgery, was defined as non-union [25]. We selected the International Society of Limb Salvage (ISOLS) method to determine the degree of integration of the grafts [26]. The functional results were assessed using the EQ5D score (EQ5D is an instrument that evaluates the generic quality of life) [27].

## 3. Results

The mean age of the patients was 23.6 years (18–44), the mean length of the femoral bone defect was 150 mm (100–240), the length of the vascularized contralateral harvested fibula autograft was 220 mm (150–280), and the duration of the second stage surgical reconstruction was 520 min (464–580). No fibula donor site morbidity was noted (Table 1). The four patients did not have comorbidities or risk factors for vascularized graft failure, excluding oncologic status and treatment for patients 2 and 3.

**Figure 4:**
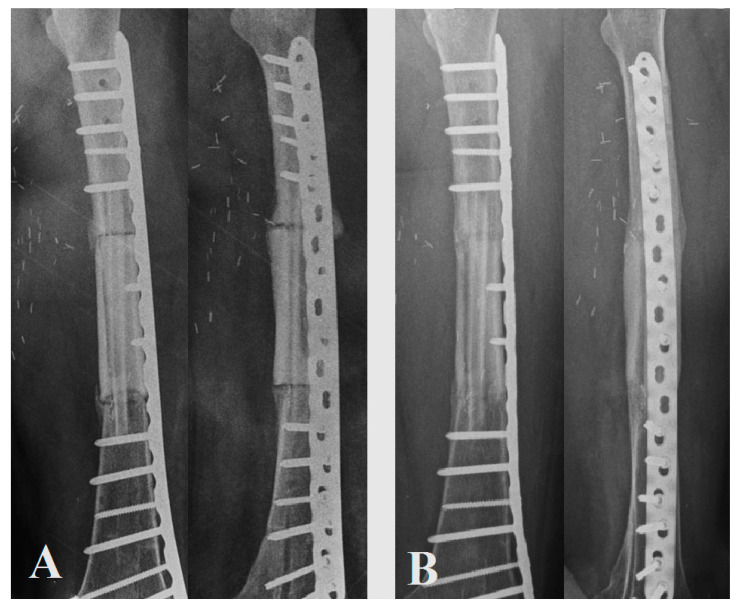
(**A**) Control X-rays for patient No. 1 at 3 months postoperatively; (**B**) control X-rays for patient No. 1 at 10 months postoperatively.

The average time between the two stages was 17 weeks (8–24). Bone union of both interfaces (defined as bridging bone across three of the four cortices evaluated at each junction in the biplane radiographs or CT scan fusion) was achieved at 5.5 months (4–7), except for one irradiated area with a patient who died of their disease. Full weight-bearing was possible at 11 weeks (8–12) in the cohort. Revision surgery was performed for one patient at three weeks for evacuation of a hematoma. Another was performed for stabilization revision (Henderson type 3) on an irradiated area; this patient died of his disease and could not achieve full bone healing due to the pronounced deterioration in their general condition. The functional scores were satisfactory with an EQ5D of 63.3 (45–75) (Figure 4). Patient No. 4 did not obtain postoperative X-rays or CT scans due to the fact of socio-financial difficulties but achieved full weight-bearing at 2 months, with no revision surgery, and was evaluated clinically.

## 4. Discussion

The preliminary results of this innovative surgical technique are encouraging, with bone consolidation being achieved within a short time, thereby allowing early full weight-bearing.

We obtained a 5.5-month (4–7) radiological bone healing time for patients with radiological follow up. This appears to be slightly better than with an isolated Capanna technique, which generally requires from six to twelve months for efficient consolidation to be obtained [4,28,29]. It also seems slightly better than an isolated Masquelet technique, which obtains full union in 4–18 months, usually on smaller tibia defects [30].

Full weight-bearing was achieved early (11 weeks (8–12)), with physiotherapy involving progressive weight-bearing, which was allowed postoperatively, while keeping the patient’s pain in mind. We assumed that fibula fusion, seen on CT scans but not necessarily on standard X-rays, may have facilitated this process. Bone healing was observed earlier on the first postoperative CT scan than on standard X-rays; CT scans performed earlier in the follow- up could have revealed better fusion times in our patients.

**Figure 5:**
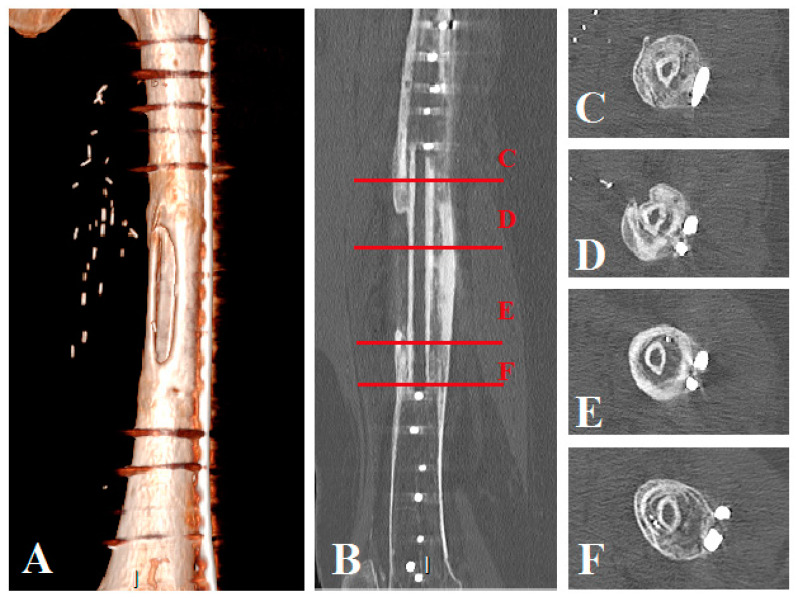
Patient No. 1 CT scan control at 14 months postoperatively: (**A**) CT scan 3D reconstruction of the healed allograft; (**B**) sagittal plane view with axial view level representation: (**C**–**F**) (**C**) axial view of the proximal host femur with the healed fibula graft, (**D**,**E**) axial views of the healed fibula graft in the allograft, (**F**) axial view of the distal host femur with the healed fibula graft.

The “Capasquelet” can be considered a method of choice for bone defects extending into the femur, particularly in complex cases requiring two-stage management; it might also be proposed in tibial resections. In the literature, the combination of several types of techniques appears to result in fewer complications, thus providing a cumulative advantage, and in tumor cases, it avoids long and harrowing one-stage surgeries [29,31,32,33,34,35]. Obtaining an osteoinductive membrane promotes bone consolidation and management of septic contexts. This osteoinductive membrane needs to be preserved for the second stage, and it should only be incised and not excised [36]. The placement of an allograft and a vascularized fibula in this induced membrane after cement removal is straightforward, with an easy workspace preserving reconstruction placement. It may allow primary (allograft compression) and secondary mechanical stability (fibula fusion) (Figure 5).

In the context of tumor resection in the case of Ewing’s sarcoma (with residual viable cells or inadequate margins), it made adjuvant radiotherapy a theoretical possibility without the risk of radiation-mediated destruction of the graft. Indeed, irradiation’s pejorative effect on bone is well described, as it causes deterioration by interfering with the trabecular architecture through increased osteoclast activity and decreased osteoblast activity [37]. With our, we might prevent the hybrid graft from these adverse events. However, the recipient bone was still subjected to irradiation, and we noted that the proximal interface did not consolidate with patient No. 3. This might be linked to irradiated recipient’s bone status, as well as due to the adverse oncological progression associated with chemotherapy and deterioration of the health status in this case.

Nonetheless, the “Capasquelet” technique also allows progressive correction of the limb length discrepancy in the first and second stages, as in the case of patient No. 2 who had a pathological fracture with recovery of 2 cm of leg discrepancy in the first stage and 2 cm in the second stage, resulting in a total correction of 4 cm. We noted two sequences of bone stability, the first obtained thanks to the allograft stabilized with a 4.5 mm LCP lateral plate, which allowed for early initiation of rehabilitation sessions and progressive weight-bearing; in the second, the vascularized fibula was able to osseointegrate as could be seen on the CT scan.

Nonetheless, with our limited follow up (as this is a new technique), these results need to be viewed with a degree of caution. Moreover, the small size of our cohort, which was mainly the result of the rarity of the indication for a two-stage reconstruction in large femoral bone defects, also needs to be taken into account. These preliminary results are of interest as, in our practice, this combination makes progressive weight-bearing possible, with full weight-bearing occurring at 3 months in complex situations, which also allows for early re-education. However, it is essential to note that this two-stage technique results in morbidity associated with the fibular harvest [38], and it requires microsurgical skills and access to a femoral allograft bank facility. The operating times involved in the two stages must also be taken into account.

## 5. Conclusions

The mean time to union in the biological reconstruction of extended bone defects varies and depends on the technique used, ranging from four to over twelve months, and full weight-bearing rarely occurs before four to six months [12,39,40,41,42]. With our method, the osteoinductive role played by the induced membrane can exert a positive impact on the bone healing of the graft, with fast allograft and fibula union, early weight-bearing, and a satisfactory functional score. This technique appears to be suitable for restoration of bone length in pathological fractures but also for preserving a hybrid graft from radiation therapy or in complex trauma cases, which are most often septic. Mid- and long-term follow up needs to be evaluated on a larger cohort with a focus on fibula behavior, allograft resorption, and functional results. Nonetheless, to our knowledge, the “Capasquelet” technique that we report here is the first to assess the combination of two main techniques for massive bone defect management, namely, the Capanna and the Masquelet techniques [11,12]. Therefore, despite its complexity, in light of the promising preliminary results with bone healing, the “Capasquelet” hybrid technique may represent a new tool for surgeons to treat critical bone defects after tumor resection or trauma in a septic context.

## Figures and Tables

**Table 1:** Summarized data for the four patients operated on using the Capasquelet technique. IMN: Intramedullary nail, T1: first stage, T2: second stage, RTA: road traffic accident, DOD: dead of disease, * Patient refused radiological follow up, with only clinical follow up.

Patient No.	1	2	3	4
Epidemiology data				
Gender	Female	Male	Male	Male
Age (years)	44	28	18	24
BMI (kg/m^2^)	24.5	22.3	20.5	24.9
Etiology	Traumatic (RTA)	Osteosarcoma	Ewing Tumor	Traumatic (Ballistic)
Length Bone Loss (mm)	100	220	180	110 (Step-cut)
Follow up (months)	36	22	14	12
Surgical data				
First-stage associated surgery	Preparation	Resection R0	Resection R0	Sepsis treatment
Spacer stabilization	IMN	IMN	IMN	Plate
Total operative time (T1; T2) (minutes)	716 (136; 580)	774 (295; 479)	656 (192; 464)	793 (235; 558)
Fibula graft length (mm)	150	280	220	160
Graft stabilization	Plate	Plate	Plate	Platex2
Delay T1 and T2 (weeks)	8	22	24	12
Postoperative data				
Complications	Hematoma	No	Material failure	No
Delay surgical revision (weeks)	3 (after T2)		28 (after T2)	
Type of surgery	Hematoma evacuation		Fixation revision	
Time for consolidation (months)	4	7	DOD	No X-ray *
ISOLS score at three months (%)	86.7	73.3	75.6	
ISOLS score at six months (%)	95.6	95.6	68.9	
Functional results				
Full weight-bearing (weeks)	12	12	12	8
EQ5D	70	75	DOD	45

## Data Availability

The data sets generated and/or analyzed during the current study are available from the corresponding author upon reasonable request.
